# Prevalence in running events and running performance of endurance runners following a vegetarian or vegan diet compared to non-vegetarian endurance runners: the NURMI Study

**DOI:** 10.1186/s40064-016-2126-4

**Published:** 2016-04-14

**Authors:** Katharina Wirnitzer, Tom Seyfart, Claus Leitzmann, Markus Keller, Gerold Wirnitzer, Christoph Lechleitner, Christoph Alexander Rüst, Thomas Rosemann, Beat Knechtle

**Affiliations:** Pädagogische Hochschule Tirol, Pastorstraße 7, 6010 Innsbruck, Austria; Medical Department, Ernst-Moritz-Arndt University of Greifswald, Fleischmannstraße 8, 17475 Greifswald, Germany; Institute of Nutrition, University of Giessen, Wilhelmstr. 20, 35392 Giessen, Germany; Institut für alternative und nachhaltige Ernährung, Am Lohacker 2, 35444 Biebertal/Giessen, Germany; AdventureV & change2V, Berchat 302/2, 6135 Stans, Austria; ITEG, Conradstraße 5, 6020 Innsbruck, Austria; Institute of Primary Care, University of Zurich, Pestalozzistrasse 24, 8091 Zurich, Switzerland; Facharzt FMH für Allgemeinmedizin, Gesundheitszentrum St. Gallen, Vadianstrasse 26, 9001 St. Gallen, Switzerland

**Keywords:** Diet, Nutrition, Running performance, Marathon, Endurance running, Vegetarian

## Abstract

**Background:**

Beneficial and detrimental effects of various vegetarian and vegan diets on the health status are well known. Considering the growing background numbers of vegetarians and vegans, the number of vegetarian and vegan runners is likely to rise, too. Therefore, the Nutrition and Running High Mileage (NURMI) Study was designed as a comparative study to investigate the prevalence of omnivores, vegetarians, and vegans in running events and to detect potential differences in running performance comparing these three subgroups.

**Methods/design:**

The NURMI Study will be conducted in three steps following a cross-sectional design. Step 1 will determine epidemiological aspects of endurance runners (any distance) using a short standardized questionnaire. Step 2 will investigate dietary habits and running history from eligible participants (capable of running a half-marathon at least) using an extended standardized questionnaire. Step 3 will collect data after a running event on finishing time and final ranking as well as a post-race rating of perceived exertion, mood status, nutrient and fluid intake during the race.

**Discussion:**

Our study will provide a major contribution to overcome the lack of data on the prevalence and running performance of vegetarian and vegan runners in endurance running events. We estimate the prevalence of vegetarians and vegans participating in a running event to be less compared to the respective proportion of vegetarians and vegans to the general population. Furthermore we will validate the subject’s self-assessment of their respective diet. This comparative study may identify possible effects of dietary behavior on running performance und may detect possible differences between the respective subgroups: omnivorous, vegetarian and vegan runners.

*Trial registration* Current controlled trials, ISRCTN73074080

## Background

The Academy of Nutrition and Dietetics (formerly the American Dietetic Association) publishes position papers to vegetarian diets since 1980. The position paper of 2009 states that well-planned vegetarian diets, including total vegetarian or vegan diets, are appropriate for individuals during all stages of the lifecycle, including pregnancy, lactation, infancy, childhood, and adolescence, and for athletes (Craig and Mangels 2009).

The current position paper states that well-designed vegetarian diets, that may include fortified foods or supplements, meet current nutrient recommendations and are appropriate for all stages of the life cycle, including pregnancy, lactation, infancy, childhood, and adolescence (Cullum-Dugan and Pawlak 2015).

Numerous reputable studies (e.g. EPIC Oxford, Adventist health Study 1&2, and GEICO Study) described distinct advantages of vegetarian or vegan diets compared to diets containing meat and other foods of animal origin (Appleby et al. 1999; Davey et al. 2003; Le and Sabaté 2014; Mishra et al. 2013; Tonstad et al. 2013).

Health related benefits of vegetarian diets range from lower mortality from all-causes (Li 2014; Orlich et al. 2013) to lower body weight (BW), Body-Mass-Index (BMI) (Williams 1997), blood pressure (Yang et al. 2011), risk of obesity, and incidence of type 2 diabetes (Chiu et al. 2014; Zhang et al. 2010), as well as an enhanced antioxidant status capable of reducing exercise-induced oxidative stress (Kim et al. 2012; Trapp et al. 2010). Generally, the health of vegetarians is sound and compares favorably with that of the non-vegetarians (Appleby et al. 1999; Deriemaeker et al. 2011). As for all kinds of nutrition schemes, the health of vegans depends on their knowledge of how to compose and appropriately supplement their diet (Gilsing et al. 2010; Key et al. 2006; Le and Sabaté 2014; Obersby et al. 2013).

Since vegetarians consume widely divergent diets, a differentiation between various types of vegetarian diets is necessary. Many misunderstandings concerning vegetarianism are due to scientific data from studies without this differentiation. In the past, vegetarian or vegan diets have been described as being deficient in several nutrients including vitamin B_12_ (Gilsing et al. 2010; Obersby et al. 2013) iron, zinc, calcium, omega-3 fatty acids and iodine (Key et al. 2006). Numerous studies have demonstrated that the observed deficiencies are usually due to poor meal planning (Leitzmann 2005).

Although there has been some concern about protein intake for vegetarian and vegan athletes, data indicate that all essential and non-essential amino acids can be supplied by plant food sources alone as long as a variety of foods are consumed and the energy intake is adequate (Nieman 1999). Furthermore well planned, varied, appropriately supplemented vegetarian and vegan diets high in nutrient density appear to successfully and effectively support parameters that influence athletic performance (Rodriguez et al. 2009), nutritional requirements, recovery and resistance to illness in athletes (Barr and Rideout 2004; Eisinger et al. 1994; Fuhrman and Ferreri 2010). Yet, some athletes, regardless of diet, may lack nutritional knowledge critical to preventing nutrition-related health problems. However, most athletes have positive attitudes toward nutrition and are receptive to nutritional counseling (Zawila et al. 2003).

Considering the growing background numbers of vegetarians and vegans (Stahler 2011; Vegetarierbund Deutschland 2015), we assume that the number counts of vegetarian and vegan runners is rising, too. Among Europeans, 5 % of the population are estimated vegetarian or vegan adding up to 37 million vegetarians and vegans overall (European Vegetarian Union 2015). Considering German speaking European countries (Austria, Germany, and Switzerland), 9 % of the Austrian population or 760,000 are estimated vegetarian and 80,000 vegan (Hnat 2015). In Germany 10 % of the population or 7.8 million are estimated vegetarian and 1.1 % or 900,000 vegan (Vegetarierbund Deutschland 2015). 5 % of the Swiss population is estimated vegetarian (Schweizerische Vereinigung für Vegetarismus 2015).

There are many vegetarian and vegan athletes especially in endurance and ultra-endurance disciplines such as Alan Murray and Janette Murray-Wakelin (marathon running) (Murray and Murray-Wakelin 2015), Michael Arnstein (marathon running) (Arnstein 2015), Fiona Oakes (marathon running), Vlad Ixel (ultra-marathon running), Scott Jurek (ultra-marathon running), Ruth Heidrich (triathlon) (Greatveganathletes.com 2015), Emil Voigt (Olympic track and field), Edwin Moses (Olympic track and field), Paavo Nurmi (Olympic track and field) (Finn 2015), Brendan Brazier (triathlon) (Braizer 2015), and Arnold Wiegand (ultra-triathlon) (Wiegand 2015).

Considering these and numerous other successful (ultra-) endurance athletes adhering to various vegetarian and vegan diets provides sufficient evidence that high-level endurance and ultra-endurance performance can be achieved by following a vegetarian or vegan diet. Therefore it is reasonable to conclude that a vegetarian or vegan diet is compatible with successful endurance and ultra-endurance performance. Yet the prevalence of vegetarians or vegans in endurance running events has not been investigated to date.

Scientific data about endurance and ultra-endurance athletes following a vegan diet is limited. Only two case reports describing vegan diet and athletic performance can actually be found in PubMed database (MEDLINE Database 2015). Wirnitzer and Kornexl (2014) described the successful implementation of a vegan diet during an 8-day mountain bike race of a female athlete. Leischik and Spelsberg (2014) investigated an ultra-endurance triathlete on a raw vegan diet, with reference to his ability to perform, cardiac status, and any symptoms of deficiency.

Since case reports are not adequate to draw any scientific conclusions to a larger population, the NURMI Study was designed as a comparative study with a large study population to investigate the prevalence in running events and to detect potential differences in running performance comparing three subgroups: omnivorous, vegetarian and vegan endurance runners.

The future results of the NURMI Study might be useful to develop adequate strategies on nutrition behavior and might help to design individualized interventions in order to meet the nutritional challenges of demanding endurance running races and the requirements of vegetarian and vegan endurance runners.

### Trial objectives

1. The first and major goal of the NURMI Study is to compare endurance performance of vegetarian and vegan runners to non-vegetarian marathon runners and to determine potential differences/to identify possible correlations.
2. The second goal is to investigate the prevalence of vegetarian and vegan runners in endurance (half-marathon and marathon) events.
3. The third goal is to validate the subjects self-reports on his/her dietary behavior.

## Methods

**Fig. 1:**
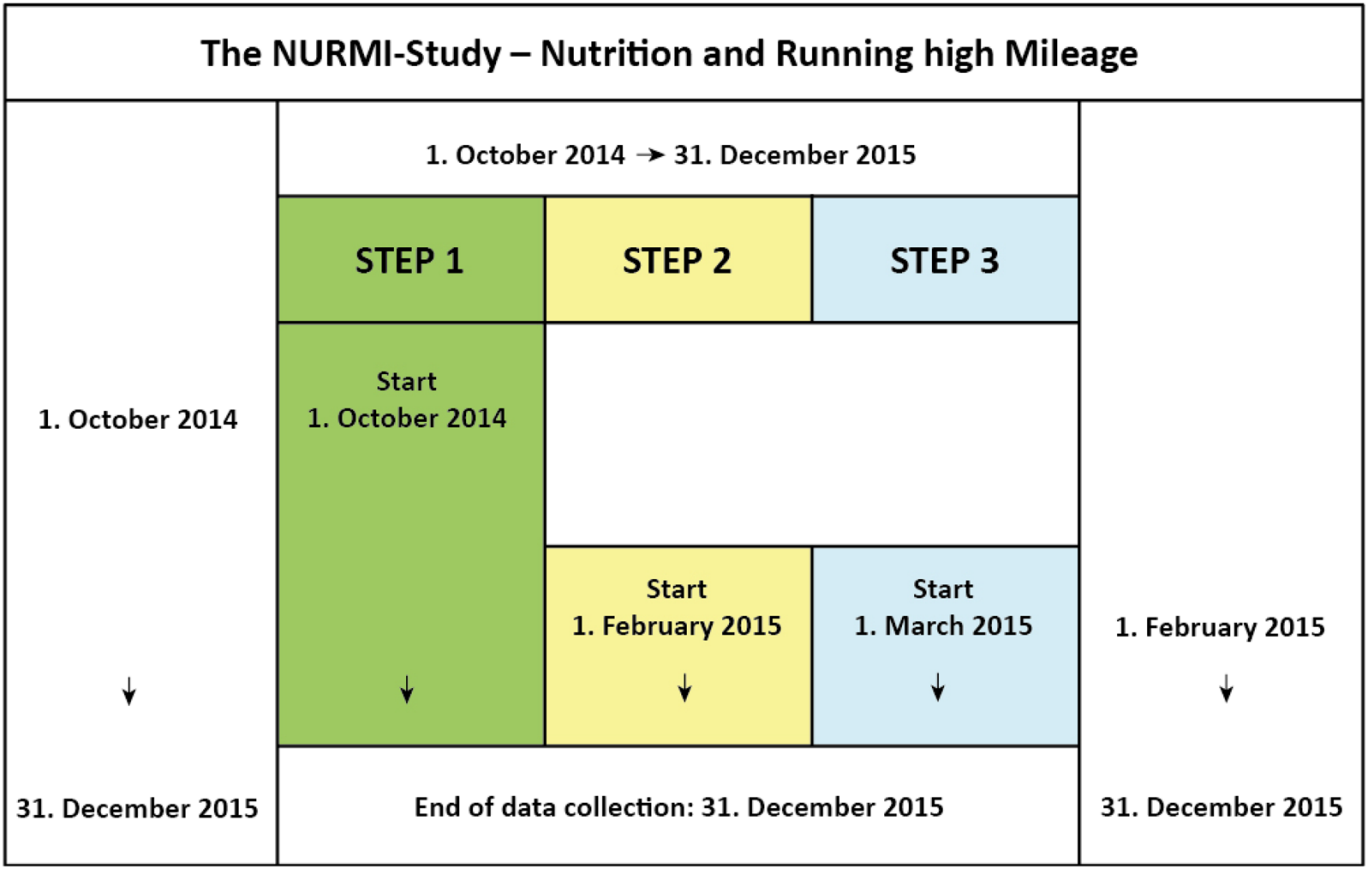
Timescale of the NURMI Study

The NURMI Study was designed by an interdisciplinary team of primary care physicians, sports medicine specialists, sport scientists, and nutrition scientists. An overview of all components of the intervention is shown in Fig. 1.

### Step 1

Epidemiological pre-study

Step 1 is composed of a short standardized online questionnaire for endurance and ultra-endurance runners (any distance and any level of performance) primarily in Austria, Germany and Switzerland. Data on sex, age, kind of diet, and running history and habits will be collected. We aim to approach as many runners as possible for this step since gathered data will be used to estimate the prevalence of vegetarians and vegans active in endurance running.

### Step 2

The major NURMI Study questionnaire

Runners matching inclusion criteria of the respective subgroups (capable of running a half-marathon etc./see “Subjects” section) complete an extended questionnaire on health status, specific dietary habits (details of consumption of specific foods or food groups), running history (i.e. preferred running distance, finished races overall and number of completed races in the last 2 years, preparation for a specific event, average distance and training load per day/week, etc.) and exercise induced diet (i.e. during training, racing or a day of rest, nutritional strategy on race day, etc.) as well as quality of life and health-related behavior. Step 2 includes a short form of the Quality of Life questionnaire (QOL BREF 26) distributed by the World Health Organization.

### Step 3

NURMI post-race testing

After individual finishing a race of at least half-marathon distance, runners complete a questionnaire on final ranking, finishing time, energy and fluid intake, in race dietary strategy, and physical and mental perception. The rate of perceived exertion (RPE, overall, leg muscles and respiratory system) will be assessed by using the Borg Scale (Borg 1998).

### Subjects

A large number of runners is planned to be enrolled in order to reach a sufficient number of subjects especially for Step 1. For Step 2 and 3 runners will need to meet the inclusion criteria. Runners participating in the NURMI Study will be recruited via advertisement by various channels such as directly at the websites of the organizers of marathon events, online running communities, and specific mailing-lists for runners, runners’ magazines as well as magazines for health, vegetarian nutrition and lifestyle and personal communication. Additionally, information will also be provided by the official website (www.nurmi-study.com). Core region are German-speaking countries of Europe (Germany, Austria, Switzerland).

#### Inclusion criteria

For the epidemiological pre-study (Step 1) any subject active in running (any distance as well as any performance level) can participate. For successful participation in the main study (Step 2 and 3) a complete data set consisting of the following four items is required: written informed consent (1), at least 18 years of age (2), all NURMI questionnaires completed (3), successful participation in a running event of either half-marathon or marathon distance (4).

### Description of measurements/methods/data collection

#### Questionnaires

All questionnaires are standardized, based on self-report, and will be conducted as online-surveys. Subjects can access www.nurmi-study.com and complete all questionnaires via encrypted interface.

#### Further measurements

Individual finishing time will be measured by the running events professional timing system and will be calculated as percentage of the overall winner’s time.

#### Voluntary physical examination (optional)

Study participation does not involve any specific invasive type of intervention. Yet, we strongly advise the subjects to undergo a medical checkup including both a blood test and an incremental exercise test on a treadmill prior to the running event. Both can be conducted by a physician and analysed at a professional diagnostics laboratory at the runners hometown to determine critical micronutrient levels of iron, haemoglobin, serum ferritin, haematocrit, magnesium, serum vitamin B_12_, holo transcobalamin, homocysteine and zinc, as well as to determine laboratory running performance. Participants will need to pay full cost for this voluntary laboratory testing, since it is optional. Subjects will be asked to measure body weight before and after the running event chosen for Step 3.

### Outcomes

#### Primary outcomes

In addition to running performance (individual marathon finishing time of Step 3) adjusted to performance level the primary outcome is the prevalence of kinds of diets (omnivorous, vegetarian, vegan) among runners attending running events.

#### Secondary outcomes

Outcomes from Step 1 are: nationality, sex, age, body weight, height, calculated BMI, kind of diet, attended races in past 2 years, finished running distances, personal best time on each distance, number of planned events for 2014/2015, in-race food and fluid consumption, daily/weekly training frequency, daily/weekly training load, period of preparation for main event, and aim of race participation.

Outcomes from Step 2 are: years of running experience, motivation for running then and now, assisted training, years of race experience, training intensity, medium/long-term goal of racing, additional specific kinds of training, specific diet including supplements, specific training/diet prior to race, diet on day of rest/training/race, pre/in/post-race diet, specific gear, relevant medical measures, and quality of life and health-related behavior.

Outcomes from Step 3 are: Pre-race: body weight (including clothing and shoes). In-race: individual finishing time, individual runtime as % of overall winners time (calculated), ranking, calculated pace, temperature, wind, air pressure, humidity, sunlight. Post-race: body weight (including clothing and shoes), calculated weight loss, RPE (Borg) whole body/respiratory/legs, mental mood, fluid and nutrient intake including breakfast, and dietary strategy during race.

### Strengths of the study

Since scientific data about endurance and ultra-endurance athletes following a vegan diet is limited the NURMI Study will be the first study to assess this issue considering a bigger sample size. The NURMI Study aims to provide a large data set comparing respective subgroups of omnivorous, vegetarian and vegan runners ranging from epidemiological aspects, dietary and training habits, in-race dietary consumption, and performance measurements. Large numbers of participants will allow to discriminate between and, identify associations with, different dietary patterns and level of training status as well as to detect differences in running performance between subgroups of omnivores, vegetarians and vegans. To make sure to assess fit runners only the NURMI Study focuses on runners who are at least capable of coping the half-marathon distance as the primary inclusion criteria.

### Limitations of the study

Not all vegetarians and vegans participating in running events may be within the reach of the various recruitment methods. Due to various season, daytime and location of races, environment conditions (e.g. weather including sun or rain, temperature, relative humidity) will vary among the respective running events. Furthermore, our study shares with others the limitation of the cross-sectional design; therefore the present investigation allows no conclusion regarding causality. Prospective cohort studies are needed to confirm the associations between specific kinds of diet, health status and endurance performance to assess the causal direction and to develop recommendations for nutritional intake for vegetarian and vegan runners for training and racing.

### Statistical methods

Analyses will be performed using commercially available software (IBM SPSS Statistics 23, SPSS Inc., Chicago, IL, USA). All data derived from the statistical methods will be given in mean ± standard deviation. Research shows that the most successful athletes in marathon and ultra-marathon running events are frequently those who lose substantially more than 3–4 % BW during competition (Zouhal et al. 2011). Mean body weight of male recreational marathon runners is 73.9 ± 8.1 kg (Barandun et al. 2012) and 75.8 ± 8.6 kg (Friedrich et al. 2014) of male recreational half marathon runners. Mean body weight of female recreational runners is 59.1 ± 6.3 kg (Rüst et al. 2013) and 60.1 ± 7.8 kg (Friedrich et al. 2014) of female recreational half marathon runners. Expected body weight loss during a marathon race is 2.3 ± 2.2 % overall (Zouhal et al. 2011). For male recreational marathon runners we expect a 2.3 % weight loss from 73.9 to 72.2 kg, therefore a sample size of 179 runners is needed to reach 80 % power with two-sided test and alpha of 0.05. For female recreational marathon runners we expect a 2.3 % weight loss from 59.1 to 57.7 kg, therefore a sample size of 159 runners is needed to reach 80 % power with two-sided test and alpha of 0.05. Since body weight loss during a marathon is inversely related to race finishing time (Zouhal et al. 2011) we expect the fastest runners to lose the most relative body weight during competition. Multi-variant regression analyses will be used the determine effects of kind of diet, sex, age, BMI, finished running distances, years of race experience, weekly training load, weekly training frequency, training intensity, in-race food and fluid consumption on individual finishing time (% of overall winners time, ranking, calculated pace). An analysis of variance will be performed to compare individual finishing time (% of overall winners time, ranking, calculated pace), sex, age, body weight, height, BMI, attended running events in past 2 years, finished running distances, personal best time on each distance, number of planned events for 2014/2015, daily/weekly training frequency, daily/weekly training load, period of preparation for main event, aim of race participation, years of running experience, motivation for running then and now, assisted training, years of race experience, training intensity, medium/long-term goal of racing, additional specific kinds of training, specific diet including supplements, specific training/diet prior to race, diet on day of rest/training/race, pre/in/post-race diet, in-race food and fluid consumption, specific gear, relevant medical measures, pre/post-race body weight (including clothing and shoes), calculated weight loss, in-race: nutritional strategy during race, fluid and nutrient intake including breakfast, mood status, and RPE (Borg) whole body/respiratory/legs, among the three subgroups created respective to diet.

### Schedule of the study

Step 1 is accessible since October 1st 2014. Step 2 is accessible since February 1st 2015 and Step 3 is accessible since March 1st 2015. All questionnaires will be accessible through December 31st 2015. Analysis and interpretation will be taking place subsequently to the study.

### Study centers

Data of all steps of the NURMI Study will be collected and analyzed in Austria. Core region are German-speaking countries of Europe (Germany, Austria, Switzerland).

### Ethical principles

The study is conducted in accordance with medical professional codex and the Helsinki Declaration as of 1996 as well as Data Security Laws and good clinical practice guidelines. Study participation is voluntary and can be cancelled at any time without provision of reasons and without negative consequences.

#### The subject’s written informed consent

Previous to study, subjects participating in the NURMI Study give written information about the content and extent of the planned procedure of the study. In case of study discontinuation, all data sets will be deleted.

#### Vote of the ethics committee

The study protocol was approved by the ethics board of St. Gallen, Switzerland on May 6, 2015 (EKSG 14/145).

#### Duties on the part of the investigators

The authors hereby confirm that ethical and scientific criteria as well as quality standards in terms of planning, study procedure, monitoring, analysis and documentation of the study will be fully observed and carried out in accordance with the protocol. All rights of the subjects will be respected and the results of the study will be handled correctly. The investigators are bound to conduct the study according to the study protocol and to report and document deviations to the ethics committee.

#### Evaluation of the risk–benefit ratio

All planned measurements are routine, justifiable and reasonable from a medical point of view. Study participation implicates no additional risk for the subject. Participation is voluntary and discontinuation will be possible at any time and without negative consequences. The study will be considered a contribution for endurance athletes, in particular endurance runners following some kind of vegetarian diet including the vegan diet. Moreover, the study aims to add knowledge to the currently very limited body of science considering the vegetarian but specifically the vegan endurance athlete. Furthermore the study could help to eliminate remaining concerns of coaches and runners and has the potential to show the adequacy of vegetarian and vegan diets on running performance as seen in professional runners.

#### Benefits for the subjects

Subjects will not be given any financial compensation. Subjects will receive a brief summary of the results of the NURMI Study if desired.

### Data security

#### IT-team

All data are treated according to appropriate Federal Data Security Laws. The NURMI online surveys hosted on a dedicated virtual server, and run https-only, therefore all relevant data are transmitted SSL-encrypted. Access to the server as well as the file- and database-backups is restricted to the IT staff of the study team and the hosting provider (also a project partner). Security measurements include a local firewall on the server itself, regular security updates of the operating system and applications, no FTP access and no unencrypted access at all. SSH access is restricted to SSH key authentication, ruling out dictionary attacks, automatic detection of dictionary and other brute force attacks against SSH, with automatic locking of attacking clients’ IP addresses, and basic intrusion detection regarding operating system and applications. Gathered data will be stored pseudo-anonymised. Each subject will be assigned an identification code (ID), which will be kept in a separate database. Questionnaire data and subject’s registration data will be stored in different databases. ID linkage will allow us to assign questionnaire data to each subject’s data set. All members of the research staff are bound to their professional obligation to discretion. Data will be used and analyzed exclusively and only in the context of the NURMI Study.

### Trial status

All questionnaires are open and will be accessible through December 31st 2015.

## Abbreviations

NURMI: Nutrition and Running High Mileage
BW: body weight
BMI: body mass index
RPE: rate of perceived exertion
IT: information technology
ID: identification code
